# Factors affecting the growth, antioxidant potential, and secondary metabolites production in hazel callus cultures

**DOI:** 10.1186/s13568-022-01449-z

**Published:** 2022-08-20

**Authors:** Roghayyeh Hazrati, Nasser Zare, Rasool Asghari-Zakaria, Parisa Sheikhzadeh, Mohammad Johari-Ahar

**Affiliations:** 1 Plant Production and Genetics, Faculty of Agriculture and Natural Resources, University of Mohaghegh Ardabili, Ardabil, Iran; 2 Department of Medicinal Chemistry, School of Pharmacy, Ardabil University of Medical Sciences, Ardabil, Iran

**Keywords:** Antioxidant enzymes, Baccatin III, Callus culture, Secondary metabolites, Sonication, Taxol

## Abstract

Hazelnut is one of the most important nut plants recently suggested as a sustainable source for paclitaxel. In the present study, the effect of the concentration and combination of PGRs, different basal medium and ultrasonic waves on callus induction and growth, physiological characteristics, and taxol and baccatin III production in hazelnut callus cultures were investigated. The results indicated that combining 2,4-D (2 mg/L) and Kin (0.2 mg/L) with the sonication of explants for 1 min provides an optimized condition for callus induction and growth. Hazelnut explants exhibited different callus production and biochemical and metabolic characteristics depending on the basal medium type, ultrasound treatment, and inclusion of ascorbic acid in the medium. So that, the highest percentage of callogenesis (100%) observed in ½ MS + 1 min US, ½ MS + 150 mg/L AA, B5 + 1 min US and B5 + 150 mg/L AA, and also ½ MS salt + Nitsch vitamins + 150 mg/L AA. Furthermore, the highest callus growth (7.86 g FW) was obtained from ½ MS + 1 min US. The highest amount of baccatin III production (147.98 and 147.85 mg/L) was obtained from the WPM and MS basal media; the highest taxol production (44.89 mg/L) was observed in the WPM basal medium. The cultures in the MS, WPM, and MS salts + Nitsch vitamins media, had the highest H_2_O_2_ and MDA content, antioxidant enzymes activity, and phenolic compounds. In conclusion, culture media nutrient composition and concentration not only affect the cell growth and physiological status of the cultures but also improve secondary metabolites production and accumulation.

**Supplementary Information:**

The online version contains supplementary material available at 10.1186/s13568-022-01449-z.

## Introduction

Medicinal plants are a valuable source of metabolites effective in the treatment of human diseases. These plants produce some complex organic chemicals called secondary metabolites or particular metabolites (Kumar and Gupta 2008). Some secondary metabolites, including taxol, have high economic value. Taxol is a diterpene alkaloid belonging to a group of diterpenes called taxane and has anticancer properties (Frense 2007). Hoffman et al. (1998) discovered and reported taxol in extracts of the hazelnut plant, and in another study, callus was induced from different hazelnut explants (Bestoso et al. 2006). In addition to plant tissues and cell cultures, hazelnut hulls and leaves and the application of different elicitors to produce taxanes have also been investigated (Safari et al. 2012; Bemani et al. 2012). Hazelnut cell extract inhibited the cell cycle of cancer cells and was more effective than Taxus extract and even pure taxol. Hazelnut extract may contain other taxol-like compounds that can enhance its anticancer effects (Bestoso et al. 2006; Bemani et al. 2012).

The development of biotechnological methods for producing secondary metabolites on a large scale is increasingly recognized as an alternative approach. Callus culture is one of the effective ways to produce high-valuable secondary metabolites (Hosseini et al. 2019). Various methods such as optimization of the medium composition, elicitors, and high throughput cell line selection can increase the production of secondary metabolites, including taxol, in in vitro cultures (Cusido et al. 2014).

Several studies have shown that various factors such as mother plant genotype, plant growth regulators (PGRs), and medium composition are significant and influential factors for in vitro growth of the plant cells (Machakova et al. 2008; Zhao et al. 2009). The composition of the medium is one of the most influential parameters for the induction of callus and the production of secondary metabolites. Most plant tissues grow in the MS medium, but due to the increasing interest in the expansion of new plant cultures, different basal media such as modified MS, B5, modified B5, Kao and Michayluk, Litvay, ER, WPM, DKW, and, SH media have been developed (Malik et al. 2011).

Most previous studies show differences in the combination and concentration of PGRs and the basal medium used to initiate and maintain hazelnut callus. MS supplemented with 2,4-D and BAP has been extensively used for hazelnut in vitro culture (Bestoso et al. 2006), and a modified B5 medium containing BAP and NAA (Bemani et al. 2012) has also been reported. Optimizing a suitable medium to obtain fast-growing in vitro cultures is essential in preparing taxanes from hazelnut callus cultures. However, no significant study has been reported on optimizing the basal medium for callus growth and taxane production in the hazelnut cultures. In the present study, besides investigating the effect of basal medium, PGRs, ultrasonic waves (Ultrasound), and casein hydrolysate on the callus induction and growth from hazelnut explants; we also investigated and compared the effect of the type of basal medium on the growth rate and metabolite (taxol and baccatin III) content in the callus cultures.

## Materials and methods

### Plant material and sterilization

In this study, fresh and mature hazelnuts were collected from the natural habitats (Fandoglu forest (latitude 38° 23′ 53.21″ N and longitude 48° 32′ 55.75″ E), Ardabil, Iran) in midsummer and the formal identification of the samples was undertaken. Experimental research, including the collection of plant material complied with relevant institutional, national, and international guidelines and legislation. The nuts were washed with running water for 20 min, soaked in ethanol (70%) for 1 min, then sterilized with sodium hypochlorite (5%) for 20 min, and finally washed three times with distilled water.

### The effect of plant growth regulators and ultrasound treatments

In order to optimize the PGRs combination and investigate the effect of US treatments, the surface-sterilized hazelnuts were divided into four parts and cultured. The explants were exposed to different durations [0 (control), 1, and 3 min] of sonication in a bath sonicator (Bandelin Sonorex Digitec, Germany) at a frequency of 35 kHz and cultured on the MS medium containing different PGRs combinations (Table 1). 30 ml of medium was used in the tissue culture glass jars and agar (8 g/L) was used to solidify the tissue culture media. The cultures were maintained in a growth chamber in the dark at 24 ± 2 °C. After 5 weeks, the callus induction and callus growth (fresh callus weight per explant) were measured.

**Table 1:** Combination of PGRs and US exposure duration used for callus induction and growth from hazelnut on MS medium

	2,4-D (mg/L)	Kin (mg/L)	BAP (mg/L)	AA (mg/L)	CH (g/L)	US (min)
1	1	0.5	–	–	–	0
2	1	0.5	–	–	–	1
3	1	0.5	–	–	–	3
4	1	0.5	–	150	1	0
5	1	–	0.5	–	–	0
6	1	–	0.5	–	–	1
7	1	–	0.5	–	–	3
8	1	–	0.5	150	1	0
9	2	0.2	–	–	–	0
10	2	0.2	–	–	–	1
11	2	0.2	–	–	–	3
12	2	0.2	–	150	1	0
13	2	–	0.2	–	–	0
14	2	–	0.2	–	–	1
15	2	–	0.2	–	–	3
16	2	–	0.2	150	1	0
17	2	–	1	–	–	0
18	2	–	1	–	–	1
19	2	–	1	–	–	3
20	2	–	1	150	1	0
21	4	1	–	–	–	0
22	4	1	–	–	–	1
23	4	1	–	–	–	3
24	4	1	–	150	1	0
25	4	–	1	–	–	0
26	4	–	1	–	–	1
27	4	–	1	–	–	3
28	4	–	1	150	1	0

### The effect of basal medium

According to the results of the first experiment, 2 mg/L 2,4-D + 0.2 mg/L Kin + 150 mg/L AA (ascorbic acid) + 1 g/L CH (casein hydrolysate) and 0 and 1 min US exposure durations were selected for investigation in different basal media (Table 2). So, the explants were exposed to 0 and 1 min US and culture on different basal media, and the percentage of callus induction, callus growth rate, secondary metabolite content, and biochemical characteristics of the calli were measured.

**Table 2:** Different basal media used for callus induction and growth from hazelnut

	Basal medium	2,4-D (mg/L)	Kin (mg/L)	US (min)	AA (mg/L)	CH (g/L)
1	MS	2	0.2	0	–	–
2	MS	2	0.2	1	–	–
3	MS	2	0.2	0	150	1
4	½ MS	2	0.2	0	–	–
5	½ MS	2	0.2	1	–	–
6	½ MS	2	0.2	0	150	1
7	B5	2	0.2	0	–	–
8	B5	2	0.2	1	–	–
9	B5	2	0.2	0	150	1
10	½ B5	2	0.2	0	–	–
11	½ B5	2	0.2	1	–	–
12	½ B5	2	0.2	0	150	1
13	WPM	2	0.2	0	–	–
14	WPM	2	0.2	1	–	–
15	WPM	2	0.2	0	150	1
16	½ WPM	2	0.2	0	–	–
17	½ WPM	2	0.2	1	–	–
18	½ WPM	2	0.2	0	150	1
19	MS salt + Nitsch vitamins	2	0.2	0	–	–
20	MS salt + Nitsch vitamins	2	0.2	1	–	–
21	MS salt + Nitsch vitamins	2	0.2	0	150	1
22	½ MS salt + Nitsch vitamins	2	0.2	0	–	–
23	½ MS salt + Nitsch vitamins	2	0.2	1	–	–
24	½ MS salt + Nitsch vitamins	2	0.2	0	150	1

### Extraction and determination of secondary metabolites

In order to prepare the methanolic extract, 0.3 g of the powdered sample was homogenized in 10 mL of 95% methanol and then shaken on a shaker at 110 rpm for 24 h at room temperature and then sonicated for 40 min. The resulting mixture was filtered through a filter and concentrated in an oven at 45 °C. The concentrated extract was dissolved in 1 mL methanol (HPLC grade) and used for phytochemical analysis and antioxidant activity assay (Chung 2019).

### Spectrophotometric determination of secondary metabolites

The total phenol content was measured using the Folin- Ciocalteu reagent according to Al-Farsi et al. (2005) with some modifications. Thus, 3 mL of diluted Folin-Ciocalteu reagent (10:1) was added to 400 μL of extract and kept in a water bath at 25 °C for 5 min. Then 3 mL of sodium bicarbonate solution (7%) was added and placed in a water bath at 25 °C for 90 min. The light absorbance of the samples was measured at 760 nm. The gallic acid standard calibration curve was used to quantify the total phenolic content. The total phenolic content was calculated in micrograms of gallic acid per gram of fresh weight.

The total flavonoid of the samples was measured according to Kumar and Sharma (2017). To one milliliter of the plant extract, 250 μL of 10% aluminum chloride solution and 250 μL of 1 M potassium acetate were added, and the absorbance of the samples was recorded at 415 nm. A quercetin standard calibration curve was used to quantify the flavonoid content in the callus samples.

### HPLC analysis of taxol and baccatin III

Separation, identification, and quantification of the amount of taxol and baccatin III were performed using the HPLC (YL9100) system and a C18 column (Eclipse XDB-C18, 5 µm, 15 × 0.46), according to Wang et al. (2016). The mobile phase consisted of acetonitrile (B) and water (A) with a multistep gradient with an injection volume of 20 μL and a 1.5 mL/min flow rate. The gradient program was 0 min, 60% (v/v) B; 0–7 min, 40–60% B; 7–7.5 min, 40–20% B; 7.5–13 min, 20% B; 13–13.5 min, 20–60% B; 13.5–19 min, 60% B. The detection wavelength for taxol and baccatin III was 227 nm. The identification and quantification of taxol and baccatin III in the callus extracts were performed by comparing their retention times (baccatin III: 1 min, taxol: 7.6 min) with those standards (Sigma-Aldrich) and using their calibration curves (Additional file 1: Figs. S1 and S2). The quantified results of taxol and baccatin III were indicated as mg/L and µg/g FW.

### Preparation of the enzymatic extract

In order for enzymatic extract preparation, 0.1 g of the powdered sample was mixed with 1.5 mL of PBS with pH = 7.8 and homogenized. The extract was centrifuged at 10,000 rpm for 20 min at 4 °C. Then, the activity of catalase (CAT), peroxidase (POD), polyphenol oxidase (PPD), and superoxide dismutase (SOD) enzymes were measured in the supernatant.

Total soluble protein concentration in the samples was measured using the Bradford method (1976). The absorbance of samples was measured at 595 nm using a spectrophotometer and quantified using the bovine serum albumin (BSA) standard curve and expressed as mg/g FW.

### Antioxidant enzymes activity assay

CAT activity was measured using one mL of reaction mixture containing PBS with pH = 7 and 30% H_2_O_2_ and 50 μL of the extract. The enzyme activity was measured as decomposition of H_2_O_2_ and decreased absorbance at 240 nm (Aebi 1984). The enzyme activity was calculated using an extinction coefficient of H_2_O_2_ (39.4 mM^−1^ cm^−1^) and expressed as mmol H_2_O_2_ decomposed mg^−1^ protein min^−1^.

POD activity was measured using one mL of reaction mixture containing 0.2% guaiacol, PBS with pH = 7, and 30% H_2_O_2_, along with 50 μL of extract. The increase in the absorbance of the samples at 470 nm was measured using a spectrophotometer (Maehly and Chance 1954). The enzyme activity was calculated using an extinction coefficient of tetraguaiacol (26.6 mM^−1^ cm^−1^) and expressed as a unit per milligram protein.

PPD activity was measured with a reaction mixture of 0.2 M Tris, 0.02 M pyrogallol, and 100 μL of the extract at a wavelength of 420 nm using the extinction coefficient pyrogallol (2.47 mM^−1^ cm^−1^). The enzyme activity was calculated and expressed as unit per milligram of protein (Kar and Mishra 1976).

SOD activity was measured by its ability to inhibit light reduction of nitroblutetrazolium (NBT) (Giannopolitis and Ries 1977). The reaction mixture contained 100 mM PBS (pH = 7.8), 1 mM EDTA-2Na, 130 mM methionine, 750 µM NBT, 20 µM riboflavin and 50 μL of the extract. The reaction was started by switching on a fluorescent lamp; it was placed under a 15-W fluorescent lamp at the height of 20 cm for 15 min, and the reaction was completed by switching off the lamps. Absorbance was measured at 560 nm, and the activity of the enzyme was calculated and expressed as a unit per milligram protein.

### Measurement of the hydrogen peroxide content

The amount of hydrogen peroxide (H_2_O_2_) was measured according to Loreto and Velikova (2001) method. For this, 0.1 g of the powdered sample was homogenized in 1.5 mL of 0.1% TCA. The samples were centrifuged for 15 min at 4 °C and 12,000 rpm. To 500 μL of the supernatant, 500 μL of 10 mM PBS (PH = 7) and 1 mL of 1 M potassium iodide were added. The concentration of H_2_O_2_ in the samples has been calculated by measuring the absorbance at 390 nm. The standard curve was prepared in the range of 2–10 mM and used to quantify the H_2_O_2_ content and was expressed as µM/g fresh weight.

### Lipid peroxidation

Lipid peroxidation level in the cell cultures was measured by the method of Heath and Packer (1968) using the extract prepared to measure H_2_O_2_. 1 mL of the reaction solution containing 20% TCA and 0.5% TBA was added to 500 μL of the supernatant. The mixture was heated at 95 °C for 30 min and immediately cooled on ice, and centrifuged. The absorbance of this solution was recorded at 532 nm 600 nm using a spectrophotometer. The amount of Malondialdehyde (MDA) was calculated and expressed as nanomoles per gram fresh weight of callus.

### Antioxidant activity assay

#### Reductive potential

According to the Chung et al. (2019) method, the reductive potential of the cell cultures has been determined. Briefly, 200 μL methanolic extract was mixed with 2.5 mL of 200 mM sodium phosphate buffer, and then 2.5 mL 1% potassium-free cyanide was added and incubated at 50 °C for 20 min. Then 2.5 mL of 10% TCA was added, and the mixture was centrifuged. To 2.5 mL of supernatant, 2.5 mL of distilled water, and 0.5 mL of 0.1% ferric chloride were added. After thorough mixing, the absorbance was measured at 700 nm.

#### DPPH free radical scavenging activity

The antioxidant activity of hazelnut cell culture extract was evaluated by measuring the reduction capacity of radicals using DPPH, according to Choochote and Suklampoo (2014). 300 μL of a one mM DPPH solution was added to 500 μL of the extract, and its volume was increased to 3 mL with methanol. After 30 min incubation in the dark, the absorbance of the solution was measured at 517 nm.

### Statistical analysis

All experiments were conducted in triplicate. Before data analysis, the normality of the data distribution was checked using a one-sample Kolmogorov–Smirnov test. Experimental data were subjected to analysis of variance (ANOVA) (p < 0.05), Pearson correlation analysis, and mean comparison using Duncan’s Multiple Range Test (DMRT) (at p < 0.05). Statistical analysis has been performed using IBM SPSS Ver.16 statistical software (IBM Corporation and Others, Armonk, NY, USA). The results were expressed as mean ± Standard error (SE) of triplicate experiments. The graphs were produced using Microsoft Office Excel 2010.

## Results

### Callus induction

#### Effect of PGRs and US on callus induction and growth

**Fig. 1:**
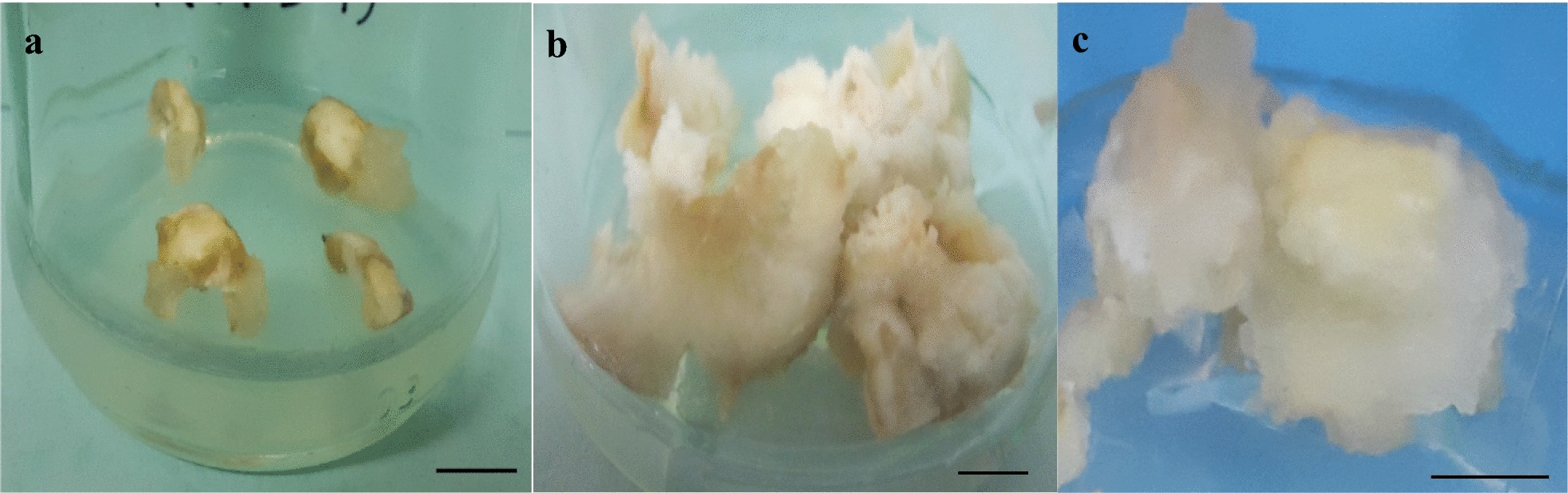
In vitro callogenesis from nut explants of *Corylus avellana*. Initiation of callus formation from explants ten days after culture (**a**); Callus growth from explants exposed to 1 min US and cultured on ½ MS medium containing 2 mg/L 2,4-D and 0.2 mg/L Kin (**b**); and B5 medium containing 2 mg/L 2,4-D and 0.2 mg/L Kin (**c**); (Scale bars = 1 cm)

**Fig. 2:**
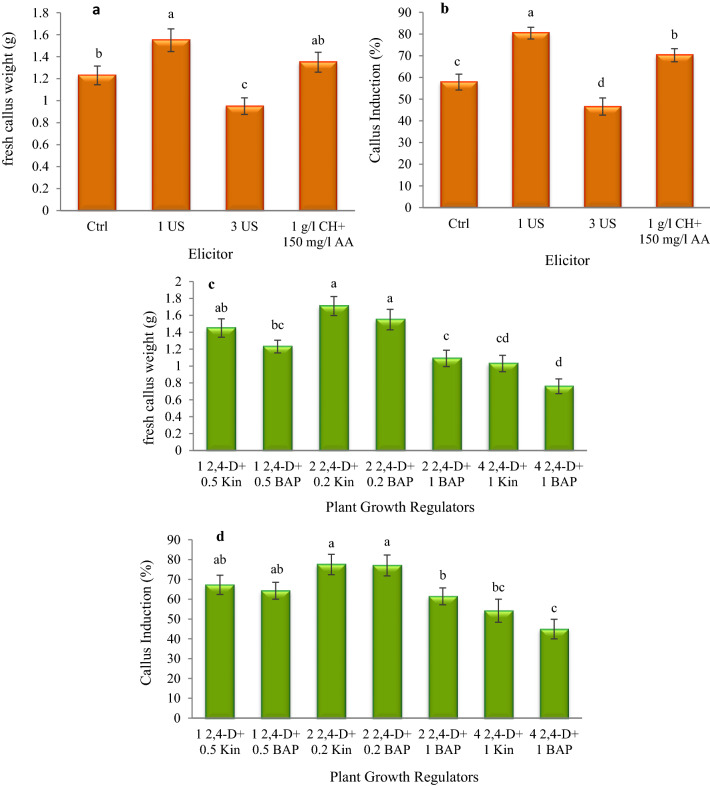
The effect of elicitors (**a**, **b**) and growth regulators (**c**, **d**) on the percentage of callogenesis and fresh callus weight. According to Duncan’s multiple range tests, values with common letters are not significantly different at the 5% probability level

In this experiment, the callogenesis of hazelnut explants was studied under the influence of different concentrations of PGRs and AA, CH, and US treatment. Furthermore, to investigate the effect of ultrasonic waves on callogenesis, some explants were sonicated (Bandelin Sonorex Digitec, Germany) at a frequency of 35 kHz for 0 (control), 1, and 3 min, and then cultured on the medium. After 10–15 days, the explants began to swell and produce callus (usually from the cut edges) (Fig. 1a). There were significant differences between different treatments in terms of the percentage of callogenesis and callus growth, but there was no significant interaction between PGR treatments and US durations. So, callogenesis percentage and callus growth varied from 25.27% and 0.49 g to 93.33% and 2.32 g depending on the type of treatment, respectively (Additional file 1: Fig. S3). In the case of US durations (elicitor), the highest percentage of callogenesis (80.45%) and fresh weight of callus (1.55 g) was observed in one minute of sonication, and the lowest percentage of callogenesis (46.58%) and fresh weight of callus (0.95 g) were observed in 3 min of sonication (Fig. 2a, b). In the case of PGRs, the highest percentage of callogenesis (77.5% and 77%) and fresh weight of callus (1.71 g and 1.55 g) was associated with 2 mg/L 2,4-D and 0.2 mg/L Kin and 2 mg/L 2,4-D and 0.2 mg/L BAP treatments, and the lowest percentage of callogenesis (44.93%) and fresh weight of callus (0.76 g) were observed in 4 mg/L 2,4-D and 1 mg/L BAP treatment (Fig. 2c, d).

#### Effect of basal medium

**Fig. 3:**
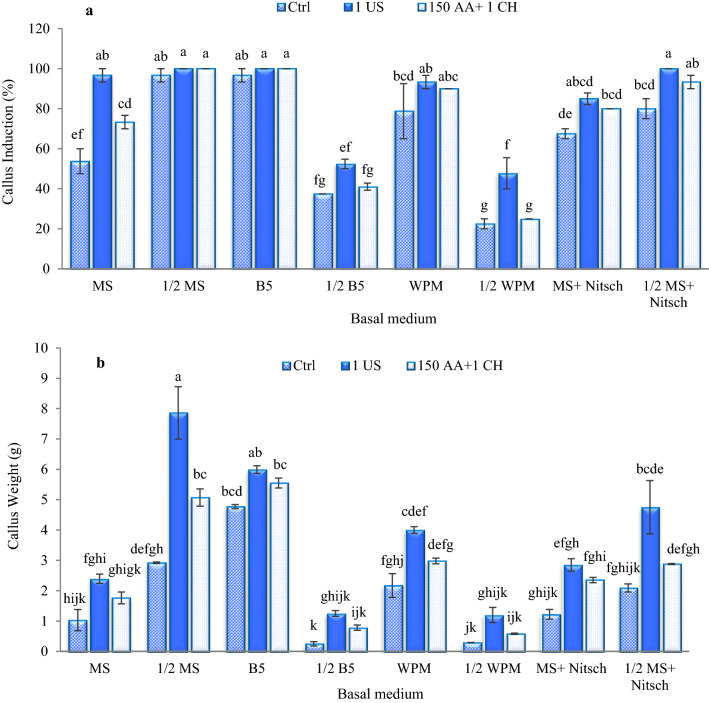
The influence of basal medium, US treatment, AA, and CH on the percentage of callogenesis (**a**) and fresh callus weight (**b**) of hazelnut explant in the medium containing 2 mg/L 2,4-D and 0.2 mg/L Kin. According to Duncan's multiple range tests, values with common letters are not significantly different at the 5% probability level

Since, in addition to various PGRs, the type and quantity of elements in the medium, i.e., macro, micro, vitamins, can affect the quality and growth of callus, it is necessary to study the appropriate medium composition. Therefore, after evaluating the callus formation results of the first experiment, the effect of different basal media on the callus induction and growth was investigated using 2 mg/L 2,4-D and 0.2 mg/L Kin, 0 and 1 min sonication, and 0 and 150 mg/L AA. The interaction between the basal medium and the elicitor was significant. The highest percentage of callogenesis (100%) was observed in ½ MS + 1 US, ½ MS + 150 AA, B5 + 1 US and B5 + 150 AA and also ½ MS salt + Nitsch vitamins + 1 US, the lowest percentage of callogenesis (22.5% and 25%) was also observed in ½ WPM and ½ WPM + 150 AA. The highest fresh callus weight (7.86 g and 5.99 g) was obtained in ½ MS + 1 US (Fig. 1b) and B5 + 1 US (Fig. 1c), and the lowest fresh callus weight (0.25, 0.29 g) in 1/2 B5 and 1/2 WPM (Fig. 3).

### Taxol and baccatin III content

**Fig. 4:**
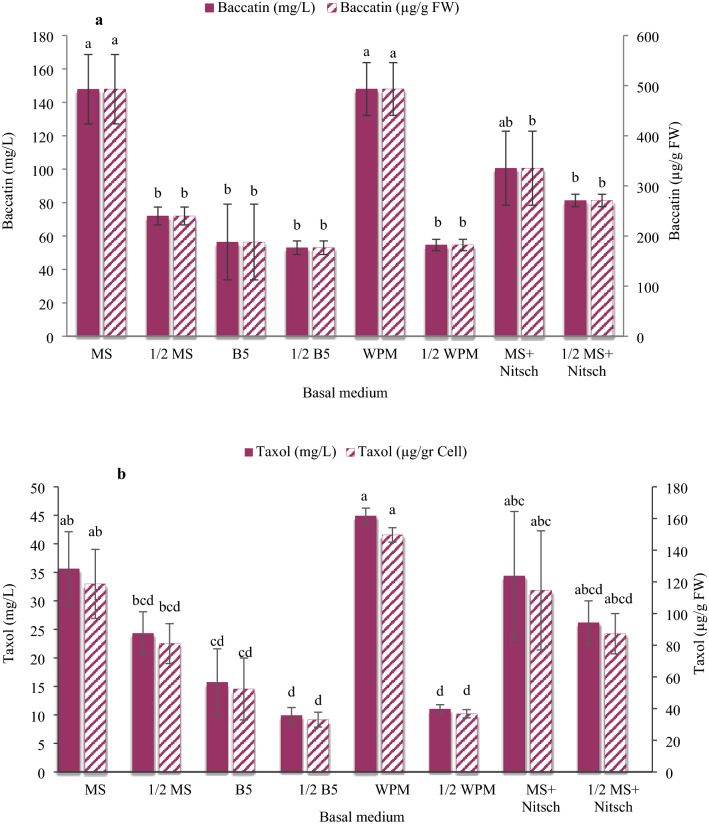
Content of baccatin III (**a**) and taxol (**b**) in the *Corylus avellana* cells cultured on the different basal media. According to Duncan's multiple range tests, values with common letters are not significantly different at the 5% probability level

Basal media differed in terms of taxol and baccatin III productivity and accumulation in the cell cultures. The highest baccatin III productivity (147.98 and 147.85 mg/L) and accumulation (493.27 and 492.84 µg/g FW) in the *C. avellana* callus cultures were obtained from the WPM and MS media, and the highest taxol production (44.89 mg/L) and accumulation (149.66 µg/g FW) were observed in WPM medium. The lowest amounts of taxol production (11.01 and 9.89 mg/L) and accumulation (36.72 and 32.99 µg/g FW) were observed in 1/2 B5 and 1/2 WPM media. The lowest amount of baccatin III productivity (53 and 54.64 mg/L) and accumulation (176.68 and 182.15 µg/g FW) were obtained from 1/2 B5 and 1/2 WPM media without significant difference with 1/2 MS, B5, or 1/2 MS + Nitsch media (Fig. 4).

### Antioxidant enzymes activity

**Fig. 5:**
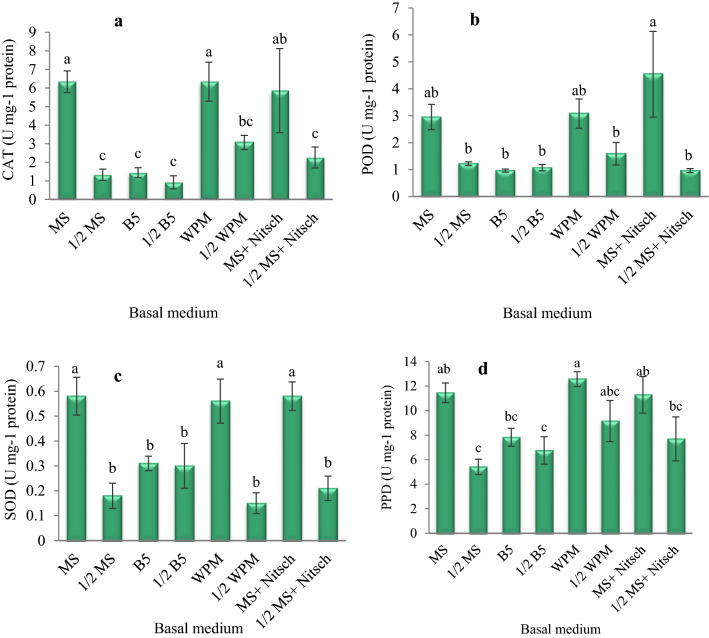
The effect of the basal medium on antioxidant enzyme activity in the *Corylus avellana* callus cultures; **a** CAT activity, **b** POD activity, **c** SOD activity, **d** PPD activity. According to Duncan's multiple range tests, values with common letters are not significantly different at the 5% probability level

According to the results, there was a significant difference between the basal medium in CAT, POD, SOD, and PPD enzymes activity. The highest CAT activity was in MS and WPM media and the following degree in MS salt + Nitsch vitamins medium. The enzyme POD was most active in MS salt + Nitsch vitamins medium, followed by WPM and MS. The highest activity of SOD and PPD enzymes was observed in the callus cultures on MS, MS salt + Nitsch vitamins, and WPM media (Fig. 5).

### The amount of H_2_O_2_ and MDA

**Fig. 6:**
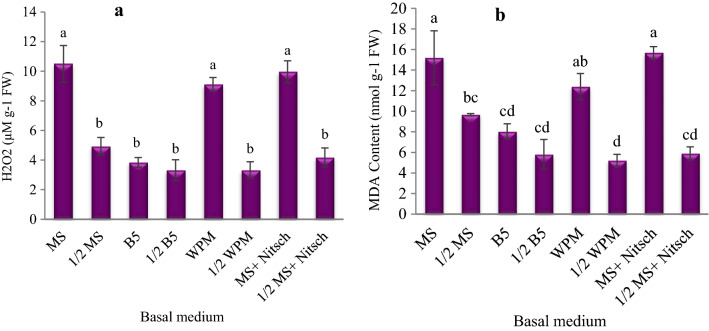
The effect of basal medium on the amount of H_2_O_2_ (**a**) and MDA (**b**) in the *Corylus avellana* callus cultures. According to Duncan's multiple range tests, values with common letters are not significantly different at the 5% probability level

The results showed a significant difference in the H_2_O_2_ and MDA content of the callus cultures between the different media. The highest amounts of H_2_O_2_ and MDA were observed in the callus cultures on the MS, MS salt + Nitsch vitamins, and WPM media (Fig. 6).

### Total phenolic and flavonoid content

**Fig. 7:**
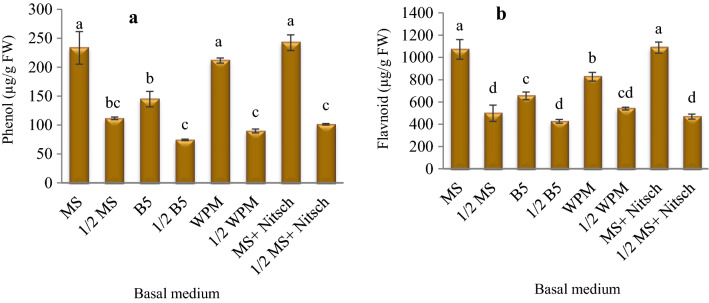
The influence of the basal medium on total phenolic (**a**) and flavonoid (**b**) content in the *Corylus avellana* callus cultures. According to Duncan's multiple range tests, values with common letters are not significantly different at the 5% probability level

There were significant differences between different basal media regarding total phenol and flavonoids. The highest amount of phenolic compounds (242.31, 233.33, and 211.54 µg/g FW) was obtained in MS salts + Nitsch vitamins, MS, and WPM media, and the highest amount of flavonoids (1088.1 and 1072.3 µg/g FW) was obtained in the MS salts + Nitsch vitamins and MS media (Fig. 7).

### Antioxidant activity

**Fig. 8:**
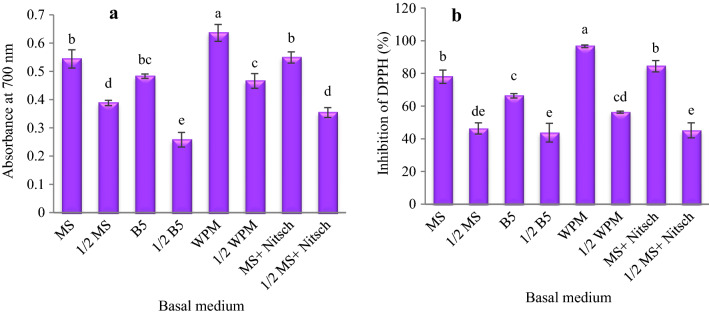
The influence of the basal medium on antioxidant activity extracts of *Corylus avellana* callus cultures; reductive potential (**a**), and DPPH radical scavenging activity (**b**). According to Duncan’s multiple range tests, values with common letters are not significantly different at the 5% probability level

In terms of antioxidant capacity, there were significant differences between different basal media. The highest reductive potential (0.636) and DPPH (96.63%) were observed in the callus cultures on the WPM medium. The lowest reductive potential (0.258) and DPPH (43.76%) were also observed in the callus cultures on the 1/2 B5 medium (Fig. 8).

A positive correlation was observed between the secondary metabolites, total flavonoids, total phenolic compounds, and antioxidant potency (Table 3).

**Table 3:**
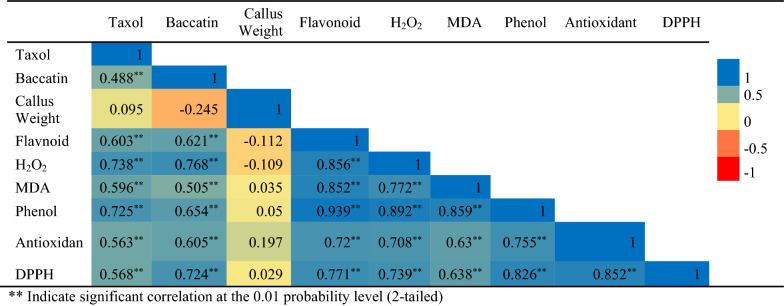
Correlation analysis between callus growth, secondary metabolites, and biochemical properties in the *Corylus avellana* callus cultures

## Discussion

PGRs are involved in all parts of the plant life cycle and profoundly affect plant cell growth, development, and responses to environmental and physiological factors and secondary metabolite production and accumulation (Richard et al. 2002). The PGRs are one of the most critical factors affecting callus formation and plant cell growth in vitro, and the type and concentration of the PGRs required for each plant or even genotype and explant should be experimentally optimized. The presence of auxins and cytokines is necessary for cell division and morphogenesis, especially during G_1_/S and G_2_/M transitions. In various plants, it has been reported that auxins are the most influential factor in the induction of callus, and cytokines support this role. Auxins increase the extensibility of the cell wall by stimulating acidification. Auxins also induce transcription of mRNAs encoding proteins involved in cell growth and development. Cytokines also directly affect the cell cycle by regulating the production of proteins involved in spindle fibers (Silveira et al. 2004). The present study and the results of other studies on yew and hazelnut cell cultures (Frense 2007; Bestoso et al. 2006) showed that auxins, especially 2,4-D, play an essential role in the stimulation of the cell division and proliferation and callus production; and its combination with cytokines such as Kin is more effective in the callus production and growth from hazelnut explants. According to the present study results, a low concentration of Kin (0.2 mg/L) performs better than high concentrations of this hormone (0.5 and 1 mg/L). In this regard, it was reported that in a medium containing 2 mg/L Kin and different auxin concentrations, either no callus produced from yew explants or the amount of produced callus is meager. However, in a medium containing 0.2 mg/L Kin, the percentage of callogenesis was very high. It was also found that in cultures containing low concentrations of Kin and high concentrations of 2,4-D, the callus cultures overgrow, and the callus structure is soft and friable, but as the concentration of Kin increases, the callus tissue also becomes stiffer (Rahmati et al. 2017).

Ascorbic acid (AA), casein hydrolysate (CH), and ultrasonic wave exposure for 1 min also positively affected callus induction and growth from hazelnut explants. It has been reported that media containing casein hydrolysates promote callus growth in various plants (Khaleda and Al-Forkan 2006). Casein hydrolysates contain various organic and inorganic materials such as calcium, phosphate, microelements, vitamins, and amino acids. Casein has been reported to promote growth in cultures where phosphate deficiency inhibits growth; hence casein hydrolysates can be considered a phosphate source (George et al. 2008). The utilization of antioxidants such as ascorbic acid in the medium composition would effectively reduce the explants and cultures’ browning phenomenon (Krishna et al. 2008). Ascorbic acid has also been shown to play some roles in plant growth, including cell division, cell wall expansion, and other growth phenomena (Piganocchi and Foyer 2003). There are several reports on the beneficial effects of ascorbic acid on various physiological and biochemical aspects of different plants (Beltagi 2008; Sheteawi 2007).

In the present study, the effect of ultrasonic waves on callus growth was positive for 1 min, but callus growth was reduced with increasing exposure duration up to 3 min. So, in media with the same PGRs composition, cultures whose explants were exposed to ultrasonic waves for 1 min and media containing ascorbic acid and casein hydrolysate had better callus growth than those that did not have these conditions. However, cultures whose explants were exposed to ultrasonic waves for 3 min had less callus induction and growth than the control. Ultrasonic waves can have different physiological and biological effects on the plant cells depending on their exposure duration and intensity. At low energy levels, US causes beneficial and reversible biological changes in cells and plant tissues. Low energy US can increase the permeability of cell membranes through the sonoporation process, which facilitates the mass transfer and molecular uptake by the cells and have a variety of biological effects on plant cells. Low intensity and energy US is of particular importance in biotechnology and has many non-destructive biological effects on living cells, including increased membrane permeability, gene expression changes, increased activity of enzymes, and levels of certain hormones (Miller et al. 1999). Wound formation in plant tissue and cells by US treatment maybe increases the indigenous hormone levels in the cells by affecting the biosynthesis and transport of plant hormones, which finally improves the callus formation and growth (Srivastava 2002). In the previous studies, it was also reported that the US at high intensities significantly reduces cell viability and survival (Safari et al. 2012; Hazrati et al. 2017). This reduction may occur due to the damages caused by the US in the cell structure, including cell membrane and wall, and organelles, which ultimately results in cell death and reduced proliferation and growth (Wu and Lin 2002).

Since callus quality and growth depend on the type and amount of nutrients (organic and inorganic compounds) in the culture medium, the basal medium composition and various PGRs can affect callus cultures’ growth and biochemical characteristics. Although MS medium containing 2,4-D and BAP has been used in hazelnut cell culture (Bestoso et al. 2006), B5 medium (Bemani et al. 2012) has also been reported. In the present study, we investigated the effect of different basal medium formulations, including MS, B5, WPM, MS salts + Nitsch vitamins, and their full and half-concentration (length) on the callus induction and growth from hazelnut explants. In general, the percentage of callus formation was more than 50% in most cultures except for ½ B5 and 1/2 WPM media, which had a lower percentage of callus formation. The highest fresh callus weight was observed in ½ MS and B5 media. MS, B5, WPM, and MS salt + Nitsch vitamins media differ in the concentration or composition of some macro and micro-elements. High concentrations or combinations of salts in the medium may affect nutrient uptake by plant cells (Mihaljevic et al. 2002). The MS and WPM medium has the highest concentration or composition of salts. Besides the differences in macronutrients, the B5 medium does not contain glycine but contains more thiamine, unlike the WPM and MS media (George et al. 2008). Plant species and even different cultivars of a given species may require different nutrients for proper in vitro culture responses. It seems that ½ MS and B5 media are better for callus growth in hazelnut in vitro cultures, maybe because these formulations have a reduced concentration of salts.

Further callogenesis in the B5 medium could also be due to the lack of glycine and the increased level of thiamine, as it has been shown that the increase in thiamine level effectively increases the callogenesis and regeneration in *Aloe vera* (Zarinpanjeh et al. 2012). Mihaljevic et al. (2002) reported that the induction and growth of yew callus are more successful in the B5 medium than in the MS medium, which reduces cell division. In in vitro culture of woody plant species, MS medium is a growth inhibitor and can be solved by reducing the ammonium concentration and completely removing nitrogen.

Typically, the optimal conditions for callus growth differ from the optimal conditions for secondary metabolite production and require careful consideration. Among the published reports on hazelnut in vitro culture, there is no report on determining the best basal medium to achieve a higher callus induction and growth rate and a more significant amount of secondary metabolites, and most studies focused on the effect of other compounds, including plant growth regulators and elicitors. In this study, some basal media were compared in terms of taxol and baccatin III production in the callus cultures of *Corylus avellana* L. The highest production and accumulation of taxol and baccatin III were obtained in the WPM and MS media. The effect of macro and micronutrients on cell growth and development is relatively straightforward and proven. Therefore, it can be concluded that these compounds can indirectly affect the biosynthesis pathways and secondary metabolites production in the cell cultures. On the other hand, many of these elements, including potassium, calcium, magnesium, and most microelements, are involved in the various cellular processes and activity of metabolic pathways. So these factors and their concentration in the culture medium can also influence the production and accumulation of secondary metabolites in the plant cells.

Vitamins in the medium composition not only influence cell growth and development but also serve as catalysts in metabolic processes. A comparison of the nature and concentration of nutrients in the used media shows that the WPM medium contains higher amounts of potassium in the form of K_2_SO_4_ than other tested basal media, as well as significant amounts of ammonium nitrate, which may directly or indirectly affect the biosynthesis pathway of taxol and other taxanes.

Furthermore, nitrogen source significantly influences the production of secondary metabolites such as terpenes and alkaloids, anthocyanins, and shikonin in the plant cell suspension cultures (Kim and Chang 1990). Ammonium nitrate is one of the most important organic compounds and can be used as a nitrogen source in many essential processes for producing secondary metabolites and the production of antioxidants (George et al. 2008). Similarly, the MS medium is high in ammonium nitrate. Ammonium readily accumulates in tissues and becomes highly toxic if not rapidly metabolized. When the ammonium concentration in the medium is low, most of the accumulated ammonium is metabolized by the cell. However, when the ammonium concentration is high, only a small amount is metabolized. The apparent difference between the B5 medium with the MS and WPM media is in their vitamin type and concentration, especially thiamine. Thiamine plays a vital role as a cofactor in the metabolic pathways of plants, such as glycolysis, the pentose phosphate pathway, and the tricarboxylic acid cycle. It has also been shown to play a cofactor role in response to biotic and abiotic stresses in plants (Goyer 2010).

The callus cultures in the MS, WPM, and MS salts + Nitsch vitamins media exhibited the higher H_2_O_2_ and MDA content and enhanced secondary metabolites production (phenolic compounds, taxol, and Baccatin III) and antioxidant enzymes activity. In contrast, the B5 medium and the 1/2 MS and 1/2 WPM media showed the lowest H_2_O_2_ and MDA content and had an inhibitory effect on the production of these compounds. There is also a positive correlation between total phenolic content and their antioxidant activity. Plants have enzymatic and non-enzymatic antioxidant defense systems to counteract the harmful effects of ROS. Enzymatic antioxidants such as SOD, POD, CAT, glutathione reductase, and ascorbate peroxidase are responsible for protecting against the toxic effects of reactive oxygen species (ROS) (Mittler 2002). The amount of ROS in a cell depends on how fast it is produced, how quickly it reacts with target molecules such as proteins, lipids, or nucleic acids, and how quickly it is broken down or neutralized by antioxidant enzymes (Mittler 2002). ROS such as H_2_O_2_ has a significant influence on cell growth and the production of secondary metabolites. MDA is a peroxidation product of unsaturated fatty acids in phospholipids. Lipid peroxidation levels have been used as a marker of free radicals level that damages the cell membranes under stress conditions (Jaleel et al. 2007). Increasing salt causes oxidative stress in cells and disrupts the physiological functions of cells. The increased MDA and H_2_O_2_ in the cell cultures on MS, MS salts + Nitsch vitamins, and WPM media may be attributed to the higher salts concentration in these basal media. Plant cell and tissue culture techniques, due to disinfection treatments, sucrose, PGRs, or the high concentration of macro-elements in the medium composition, may induce stressor conditions such as oxidative stress in the plant cells and tissues. Plant cells activate a complex adaptive mechanism for adaptation and confrontation with this stressor condition associated with epigenetic, physiological, and metabolic changes (Gaspar et al. 2002). In *Catharanthus roseus*, the utilization of high concentrations of calcium chloride reduced callus growth (Siddiqui and Mujib 2012).

The positive correlation between total flavonoids, total phenolics, and antioxidant activity indicates the role of these compounds in the antioxidant capacity of the hazelnut cells. In other words, the increased level of H_2_O_2_ in the cells activates the signaling pathway by inducing oxidative stress, which may stimulate the plant cell defense mechanisms and the production and accumulation of secondary metabolites (Wang et al. 2009). For instance, the increased level of H_2_O_2_ induces the phenylpropanoid compounds biosynthetic pathway and production of phenolic compounds in stevia under salicylic acid and methyl jasmonate treatment, maybe through increasing the transcription of phenylalanine ammonia-lyase (PAL) (Wang et al. 2009). At higher concentrations of phenolic compounds, due to the increase in the number of hydroxyl groups of the aromatic rings of the phenolic compounds in the reaction, the probability of hydrogen being given to free radicals increases, and consequently, the inhibitory power of the extract increase (Zhang et al. 2009).

Generally, it can be concluded that in addition to the appropriate concentration and combination of PGRs, the concentration and type of basal medium have a crucial effect on callus formation, the physiological status of cultures, and the production of secondary metabolites. In contrast to the callus formation and growth, which grew better in media with lower salt concentrations, the production of secondary compounds increased in the basal media with high salt content. This may be due to the oxidative stress condition induced by higher salt concentration in these basal media, which results in an increase in H_2_O_2_ production and accumulation in the cells and membrane lipid peroxidation (MDA content), consequently induction of plant cell defense responses. Furthermore, short-term exposure of the explants to ultrasound (low-energy ultrasonic waves) also positively affects callus formation.

## Supplementary Information

**Additional file 1: Fig. S1.** The retention time of the taxol and baccatin III standard in the HPLC. **Fig. S2.** HPLC chromatogram of the extract of callus. **Fig S3.** The effect of elicitors and growth regulators on the fresh callus weight (a) and percentage of callogenesis (b).

## Data Availability

All data generated or analyzed during this study are included in this published article.
