# Supplementary material for: Factors affecting the growth, antioxidant potential, and secondary metabolites production in hazel callus cultures
Source: AMB Express. 2022 Aug 20;12:109. doi: 10.1186/s13568-022-01449-z (PMC9392833; doi:10.1186/s13568-022-01449-z)
Supplement: Supplementary file 1 — Additional file 1: Fig. S1. The retention time of the taxol and baccatin III standard in the HPLC. Fig. S2. HPLC chromatogram of the extract of callus. Fig S3. The effect of elicitors and growth regulators on the fresh callus weight (a) and percentage of callogenesis (b). [file 13568_2022_1449_MOESM1_ESM.docx]

**Additional file 1**

**Factors affecting the growth, antioxidant potential, and secondary metabolites production in hazel callus cultures**

**Roghayyeh Hazrati^a^, Nasser Zare^b*^, Rasool Asghari-Zakaria^c^, Parisa Sheikhzadeh^d^, Mohammad Johari-Ahar^e^**

^a^ Plant Production and Genetics, Faculty of Agriculture and Natural Resources, University of Mohaghegh Ardabili, Ardabil, Iran. Email: [ry.hazrati@gmail.com](mailto:ry.hazrati@gmail.com); [ry.hazrati@uma.ac.ir](mailto:ry.hazrati@uma.ac.ir)

^b^ Plant Production and Genetics, Faculty of Agriculture and Natural Resources, University of Mohaghegh Ardabili, Ardabil, Iran. E-mail: [Zarenasser@yahoo.com](mailto:Zarenasser@yahoo.com); [nzare@uma.ac.ir](mailto:nzare@uma.ac.ir)

^c, d^ Plant Production and Genetics, Faculty of Agriculture and Natural Resources, University of Mohaghegh Ardabili, Ardabil, Iran. [rrasghari@yahoo.com](mailto:rrasghari@yahoo.com)

^e^ Department of Medicinal chemistry, School of Pharmacy, Ardabil University of Medical Sciences, Ardabil, Iran. [johariahar@gmail.com](mailto:johariahar@gmail.com)

*** Correspondence:** Tel: +98(45) 31505113, Fax: +98(45)33512204 E-mail: [nzare@uma.ac.ir](mailto:nzare@uma.ac.ir) , [zarenasser@yahoo.com](mailto:zarenasser@yahoo.com)


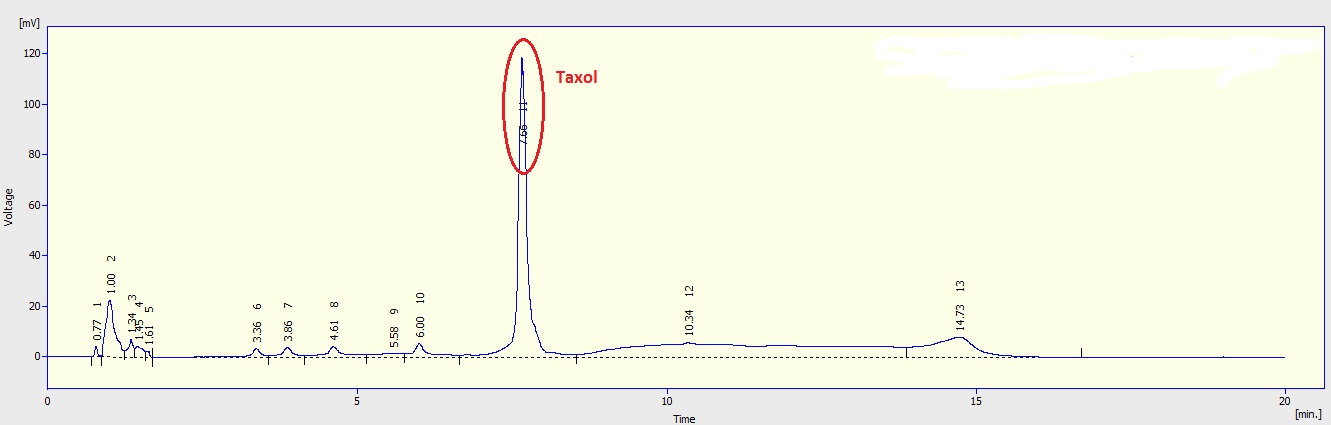


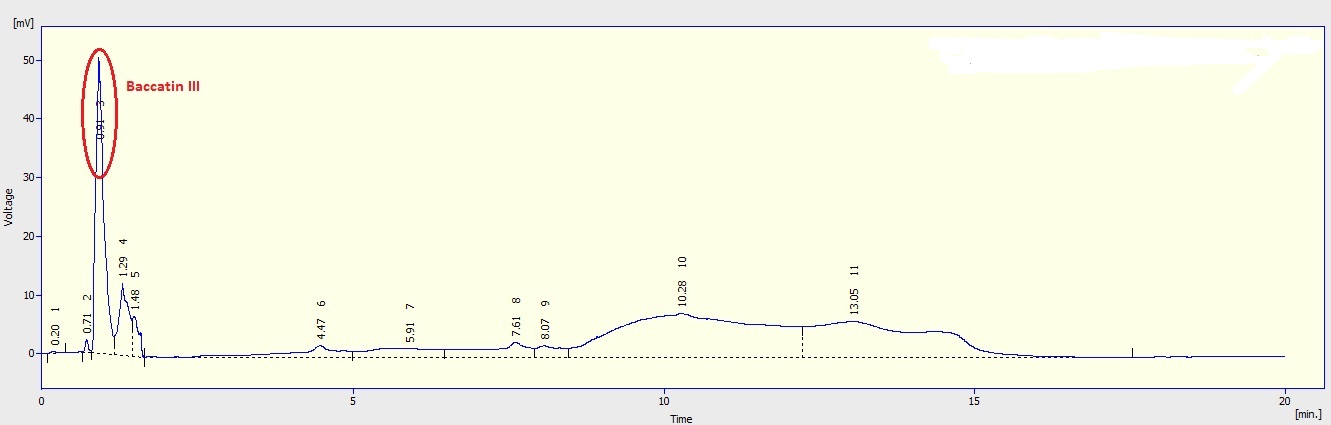


# Fig S1 The retention time of the taxol and baccatin III standard in the HPLC

#
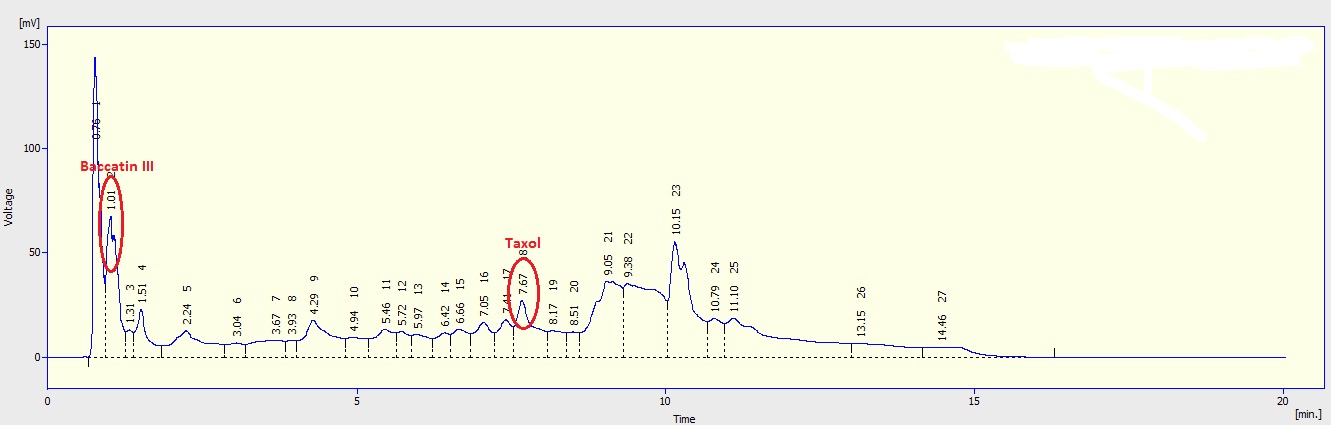
 Fig S2 HPLC chromatogram of the extract of callus

**a**

**b**

# Fig S3 The effect of elicitors and growth regulators on the fresh callus weight (a) and percentage of callogenesis (b)
